# Identification of factors affecting rice yield gap in southwest China: An experimental study

**DOI:** 10.1371/journal.pone.0206479

**Published:** 2018-11-14

**Authors:** Yuanyuan Ran, Hui Chen, Dinglun Ruan, Hongbin Liu, Shuai Wang, Xiaoping Tang, Wei Wu

**Affiliations:** 1 College of Resources and Environment, Southwest University, Chongqing, China; 2 Shapingba Meteorological Bureau, Chongqing, China; 3 Chongqing Agricultural Technology Extension Station, Chongqing, China; 4 College of Computer and Information Science, Southwest University, Chongqing, China; Tennessee State University, UNITED STATES

## Abstract

Knowledge about the relative importance of influencing-factors on rice yield gap is crucial to rice production, especially in southwestern China where topography is extremely complicated. In the current study, the data of rice yield from a total of 76 experiments were collected in 2008 and 2009 in Chongqing, southwest China. For each location, two treatments with fertilizer and without fertilizer were carried out, each treatment was performed with three replications, and yield gap was calculated using fertilized yield minus unfertilized yield. Seventeen influencing-factors including variety, fertilization, climate, terrain, and soil properties were obtained at each location. Regression tree (RT) model were employed to investigate relative important of influencing-factors to rice yield gap variability. The result of Pearson correlation analysis suggested yield gap of rice was positively correlated with sunshine hours, phosphorous and potassium fertilizers, while negatively correlated with soil available nitrogen content. The results of RT showed that the selected influencing-factors explained about 74.1% of rice yield gap variation. Meanwhile, the result also indicated variety followed by others had more influence on rice yield gap variation. Our findings analyzed by regression model at a regional scale suggested that more precise fertilization recommendation should be formulated based on comprehensive factors (e.g., soil, climate, terrain, variety), which reasonably guided farmer and government for rice production.

## Introduction

China is one of the largest rice production countries, with a total planting area of about 30.3 million hectares, accounting for about 30% of the total rice output in the world (FAO) [1]. Rice is a major grain crop after wheat and its yield is closely related to food security and sustainable development of the society. In recent years, a great deal of efforts had been conducted to keep pace with the increasing food requirement of people. However, it was generally considered that rice production was associated with massive limiting factors, such as variety, climate, terrain, and soil properties [2–4].

Previous studies reported variety updating and the improvement of management practices were contributed to increasing rice yield [5–8]. They pointed out the adoption of new variety could enhance harvest index and overcome the negative effect of climate changes. However, some studies reported climate had significant effect on rice production in the world [4,9–12]. For example, Sarker et al. [13] illustrated the temperature-related indicators (maximum temperature and minimum temperature) had more significant impact on rice yield than rainfall in Bangladesh. In India, night temperature and radiation showed significantly negative and positive influence on rice yield, respectively [14]. In China, Tao et al. [15] demonstrated appropriate temperature was positively correlated with rice yield and drought in summer was possible to decrease rice yield in Chongqing [16].

Additionally, crop could not be separated from soil nutrients to growth. Soil properties, such as available nitrogen, available phosphorus, and available potassium, exert enormous effect on crop yields. Relevant research suggested spatial variability of rice yield was mainly caused by soil chemical properties [17].

Nevertheless, soil properties and climate were closely related to terrain factors [18,19]. Thus, the variability in rice yield caused by terrain was observed [20,21]. For instance, in southwestern China, Li et al. [3] found that rice yield was strongly affected by rising elevation because of decrease accumulated temperature.

In recent years, increased rice yield resulting from application of fertilizer has been observed by long-term observational experiments [22,23]. Shrestha and Deb [24] reported fertilizer not only offset gap between yield with fertilizer and without fertilizer, but also overcame the negative effect of climate on rice growth. However, preliminary observation found that increased rice yield varied obviously among different experiments. Decision trees are gaining favor in various fields for exploring non-linear relationships between independent and dependent variables [25–30]. They are non-parametric methods and can automatically deal with both categorical and continuous variables. Decision trees are scalable to large problems and can handle smaller data set than artificial neural networks [31]. Classification and regression tree (CART) is a typical decision tree algorithm for predicting continuous variable (regression) or categorical variable (classification). A particular benefit of CART is its cross-validation feature that attempts to detect over-fitting [25]. One of the outcomes of CART is the relative importance of independent variables to the response, which could be used to investigate the factors controlling rice yield gap (fertilized yield minus unfertilized yield). Therefore, the main objectives of this study are to (1) analyze the relationship between influencing-factors and rice yield gap between fertilization and no fertilization, (2) quantify the relative importance of influencing-factors on yield gap.

## Materials and methods

### Study area

**Fig 1:**
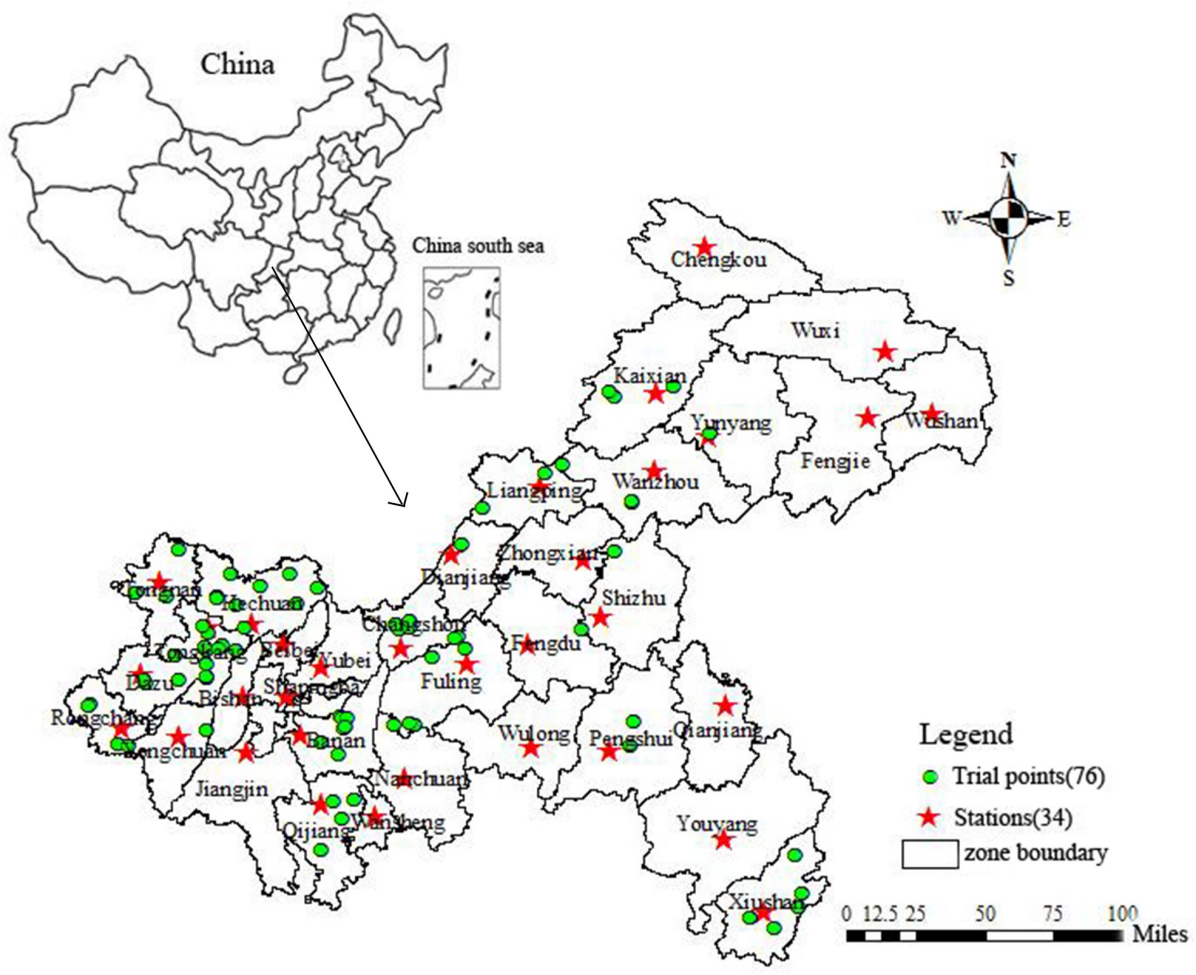
Maps study area location and trial points.

Chongqing (105°11'~110°11'E, 28°10'~32°13'N) is located in southwestern China and covers 8.24×10^4^ km^2^ (Fig 1). It is one of the most important rice production regions in China. Chongqing is characterized by hills and mountainous with elevation varying from 145 to 2763 m. The climate is moderate subtropical with striking resource superiority. The annual rainfall is about 1200 mm and mainly concentrates in April to September. Average annual sunshine hours varies from 1000 to 1650 h and temperature ranges from 6 to 38°C. Paddy soil is mostly distributed across the study area.

### Data

#### Rice yield gap

**Fig 2:**
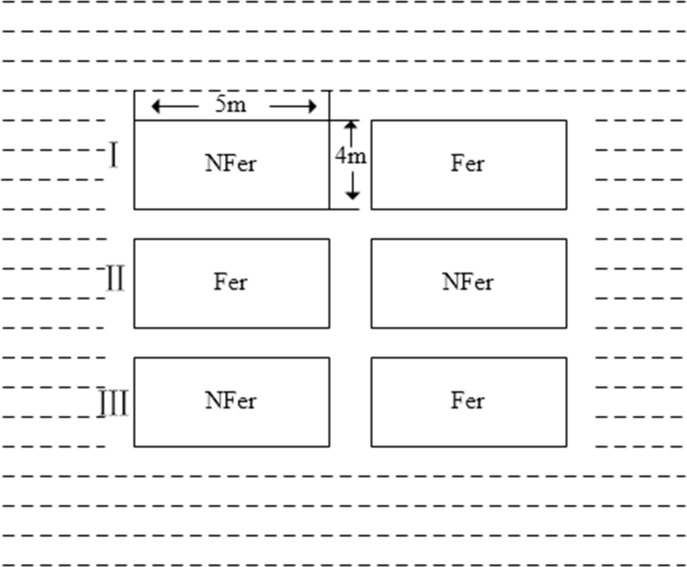
Field trials (Ⅰ, Ⅱ, Ⅲ represent three replications, respectively. Dotted line represents guarding row. NFer and Fer represent plots without fertilizer and with fertilizer, respectively).

Field trials were carried out in 2008 and 2009. The study did not involve private land, protected land, endangered or protected species. No specific permissions were required for these locations/activities. A total of 76 trial points were conducted across the study area (Fig 1). Each plot had an area of 20 m^2^ and four guarding rows around it (Fig 2). For each field trial, two treatments with fertilizer (nitrogenous, phosphorous, and potassium fertilizers) and without fertilizer were performed. In order to avoid the random effect, each treatment had three replications. Rice is usually transplanted in April and harvested in September. For each trial, rice yield was the mean of the three replications for the two scenarios. Usually, rice yield with fertilizer was higher than that of without fertilizer. Then, yield gap was calculated by fertilized yield minus unfertilized yield.

Three main hybrid rice series including Q-you (QY), Zhongyou (ZY), and Gangyou (GY) were planted widely in Chongqing. Specifically, QY series includes QY-1, 5, 6, 8, 12, 108 varieties, GY series includes GY-158, 188, 364, 615, 6366, 725, 825, 881 varieties, ZY series includes ZY-177, 36, 838, 9801 varieties. In this work, the rice varieties that the local farmers preferred were assigned to the trials (Table 1). For each trial, the two treatments were planted the same variety. The rates of fertilization recommend by Soil Testing and Formulated Project (a kind of fertilization technology in China) were summarized in Table 2, which were carried out based on absorption regularity of crop to fertilizer, the nutrient supplying capability of soil, and fertilizer use efficiency. Therefore, the difference in rates of fertilization could be found in different trials.

**Table 1:** The numbers and distributions of three series.

Series	Variety	County	Number
**QY**	QY1, QY5, QY6, QY8, QY12, QY108	Tongliang,Tongnan,Wanzhou, Yunyang,Shizhu,Pengshui, Kaixian,Hechuan,Banan,Fengdu,Yongchuan	29
**GY**	GY6366, GY158, GY188, GY364,GY615, GY725, GY825, GY881,GY177, GY363, GY527	Tongliang,Wanzhou,Rongchang, Liangping,Fuling,Dazu,Dianjiang, Tongnan,Changshou	31
**ZY**	ZY36, ZY838, ZY9801, ZY177	Xiushan,Qijiang,Kaixian, Banan,Fuling,Yunyang	16

ZY, QY, GY represent Zhongyou, Q-you, Gangyou, respectively.

**Table 2:** The rates of N, P, K fertilizer in different trial points.

N (kg/ha)	P_2_O_5_ (kg/ha)	K_2_O (kg/ha)	Number
150	90	90	57
180	90	90	5
150	72	72	7
210	90	120	1
90	45	45	4
180	120	90	1
150	45	45	1

#### Soil properties

Soil samples were collected from a depth of 0–20 cm of cultivated horizon using a manual coring tube before cultivating. At each site, 15 sub-samples were mixed in a bag, then extracted soil of about 1 kg as representative soil samples. Successively, soil samples were analyzed for chemical properties by conventional soil Agro-chemical analysis methods after air drying. Specifically, organic matter (OM), available nitrogen (AvN), available phosphorus (AvP), and available potassium (AvK) were measured by glass soil bath-K_2_Cr_2_O_7_ titration method [32], micro-diffusion method [33], Mo-Sb colorimetric method [34], flame photometer, respectively. Parent material was not included in this paper because they had no significant effect on rice growth and grain yield in our study area. Meanwhile, pH with the mean value of 6.02 also had no obvious difference due to sub-acid environment where rice was planted generally.

#### Climate variables

**Fig 3:**
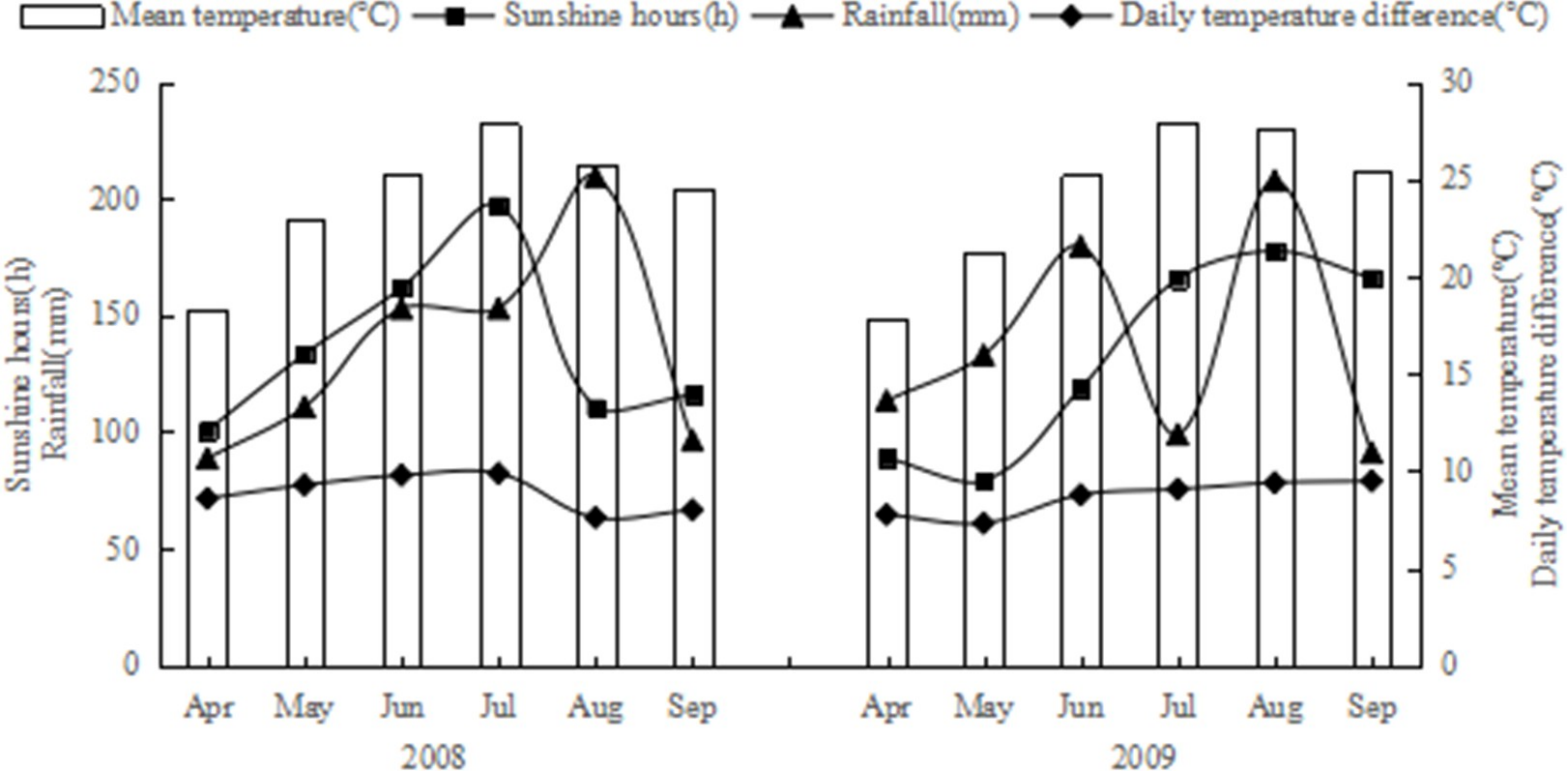
Monthly rainfall, sunshine hours, mean daily temperature and temperature difference during rice growth period in 2008 and 2009.

During the years of 2008–2009, daily climate data recorded at 34 stations in Chongqing were obtained from the National Meteorological Information Center (NMIC), China Meteorological Administration (CMA). Four climate variables, namely, mean temperature (Temp), daily temperature difference (diurnal maximum temperature minus minimum temperature, hereafter TDiff), sunshine hours (sun hours) and rainfall were calculated and used in this paper. During the rice growth period (April to September), each climate factor was examined and no missing data was found over the study area. Monthly averages for daily observed climate data during the rice growth period in 2008 and 2009 were shown in Fig 3. Monthly maxima of rainfall and sunshine hours were in August and July, respectively. There was no significant difference between 2008 and 2009 for mean temperature, mean temperature difference, and total of sunshine hours, rainfall during the growth period. Recently, thin Plate Spline (TPS) methods have been widely applied to interpolate climate parameters [35–41]. Previous works demonstrated that TPS with latitude, longitude, and elevation performed better than others, such as ordinary kriging, inverse distance weighting, multiple linear regression with latitude, longitude, and elevation plus ordinary kriging. Therefore, TPS with latitude, longitude, and elevation was applied to interpolate the climate parameters over the study area [40,41]. Then, the climate variables were extracted by the 76 trial points to analyze their effects on rice yield gap variation.

#### Topographic variables

A digital elevation model (DEM) with a spatial resolution of 30 m was used in the current study. Five commonly used terrain indicators including elevation, slope, aspect, topographic wetness index (TWI), and topographic position index (TPI) were generated from the DEM. TWI and TPI were calculated as follow:

TWI=ln(α/Tanβ) (1)

where ln indicates natural logarithm, α and Tanβ represent upslope area per unit contour length and local slope angle acting on a cell, respectively [42].

$$TPI=Z_{0}-\bar{Z}$$ (2)

$$\bar{Z}=\frac{1}{n_{R}}\sum Z_{i}\left(i\in R\right)$$ (3)

where *Z*_0_ and $\bar{z}$ represent the elevation at central point and mean elevation around it within a determinate radius (R), respectively. Positive value of TPI denotes that elevation at central point is higher than its mean surroundings and vice versa. According to Weiss [43], six topographic positions including ridge, upper slope, middle slope, flat slope, lower slope, and valley were identified across the study area.

### Methods

#### Statistical analysis

Descriptive statistics analysis was employed to examine the variation of rice yield gap and influencing-factors. Pearson correlation analysis which is a widely used method was performed to explore the relationship between rice yield gap and influencing-factors. Analysis of variance (AVOVA) combined with multiple comparisons by Tukey’s honestly significant difference was applied to test differences in yield gap and influencing-factors among the three varieties and topographic positions (QY, GY, ZY). Root mean squared error (RMSE) and mean absolute error (MAE) were used to evaluate accuracy of prediction model. All formulas were defined as:

$$r=\frac{\sum \left(f_{i}-\bar{f})(y_{i}-\bar{y}\right)}{\sqrt{\sum {\left(f_{i}-\bar{f}\right)}^{2}\sum {\left(y_{i}-\bar{y}\right)}^{2}}}$$ (4)

$$MAE=\frac{1}{N}\sum_{t=1}^{N}\left|\left(f_{i}-y_{i}\right)\right|$$ (5)

$$RMSE=\sqrt{\frac{1}{N}\sum_{t=1}^{N}{\left(y_{i}-f_{i}\right)}^{2}}$$ (6)

where f_i_ and y_i_ represent the prediction and observation (i = 1,2,3,…,76), respectively.

#### Regression tree

Regression tree (RT) which was proposed by Breiman et al. [25] is a non-parametric statistical method. RT automatically selects variables holding the most information. In regression tree, the least squared deviation (LSD) as impurity measure for splitting rules was employed, it aims to minimize intra-class variance and maximize variance among groups. Given a set of D = {(x_1_,y_1_), (x_2_,y_2_), …, (x_n_,y_n_)}, the regression model was calculated as following:

$$f(x)=\sum_{m=1}^{M}c_{m}I(x\in R_{m})$$ (7)

where R_1_, R_2_,…, R_m_ represent units which was divided, c_1_, c_2_,…c_m_ represent the fixed value outputted in each unit, respectively.

The LSD criterion function is defined as:

$$SS(t)=\sum_{i=1,Xi\in t}^{N(t)}(y_{i}-\bar{y}(t){)}^{2}$$ (8)

where t and N(t) represent node and the numbers of sample in it, respectively. y(t) represent the mean value of response variable in each node.

$$Q(s,t)=R(t)-R(t_{L})-R(t_{R})$$ (9)

where R(t_L_) and R(t_R_) represent the sum of square of left and right child node, respectively. The split node s was used to maximize Q(s,t).

RT has been applied to various fields, such as, groundwater level prediction [44], plant litter decomposition [45], as well as crop yield [30]. In the current study, RT was employed to investigate the relative importance of climate, soil properties, terrain, and management factors affecting rice yield gap variation. After several experiments, the optimal parameters were obtained by RT. Numbers of parent node and child node were 4 and 2, respectively, tree depth was 3. To avoid overfitting, the ten-fold cross-validation was applied to examine the model performance [46–48]. Samples were randomly separated into ten subsets. Each subset contains all the three varieties. The performance of RT was compared with multiple linear regression (MLR, Eq (10)).

$$y=b_{0}+\sum_{i=1}^{n}b_{i}x_{i}$$ (10)

where y was yield gap, n was the number of the independent variables (x).

All calculations were done by SPSS.19.0 and Excel 2016.

## Results

### Descriptive statistics

Descriptive statistics of yield gap and influencing-factors were summarized in Table 3. The coefficient of variation was used to examine the variability of variables. Yield gap varied from 0.2 to 4.1 t/ha, showing moderate variability with CV of 37.4%. All climate parameters presented low variability across the study area. For terrain indicators, elevation, aspect, and TWI showed moderate variability (CV = 35–64%), while slope presented strong variability. For soil properties, AvN and AvP showed the lowest and highest variability, with a changing magnitude of 24.9 mg/kg and 81.5 mg/kg, respectively. The rates of fertilizer had low variability.

**Table 3:** Descriptive statistics of influencing-factors and yield gap.

	Item	Range	Min	Max	Mean	Std.D	Skewness	Kurtosis	CV(%)
**Soil**	OM(g/kg)	39.8	3.6	43.4	24.7	8	-0.4	0.7	32.6
	AvN(mg/kg)	168	69	237	136.3	33.9	0.7	1.1	24.9
	AvP(mg/kg)	32.8	0.5	33.3	7.7	6.3	2.1	5.4	81.5
	AvK(mg/kg)	135	40	175	84	31.2	0.7	0.1	37.2
**Terrain**	Elevation(m)	671	152	823	377.7	134.2	1.4	2	35.5
	Aspect(°)	352.7	5.2	357.9	170.4	109.6	0.2	-1.2	64.3
	Slope(°)	23.5	0.3	23.9	5.6	5.3	1.9	3.8	94.9
	TWI	13.1	5.9	19	10.1	3.6	0.8	-0.5	35.1
**Climate**	Rainfall(mm)	409.6	617.9	1027.5	822.5	81.2	-0.1	-0.1	9.9
	Tmean(^o^C)	4.7	21	25.6	24	0.9	-1.3	1.8	3.7
	Sun hours(h)	333.6	636.4	969.9	779.4	68.7	0.5	0.7	8.8
	TDiff(^o^C)	1.91	7.72	9.63	8.5	0.47	0.4	0.72	5.6
**Fertilizer**	N(kg/ha)	120	90	210	150	17.7	-1.2	7.2	11.8
	P_2_O_5_(kg/ha)	75	45	120	85.8	12.6	-2.1	5.7	14.7
	K_2_O(kg/ha)	75	45	120	85.8	12.6	-2.1	5.7	14.7
**Yield**	Yield gap(t/ha)	3.9	0.2	4.1	2.2	0.82	0.08	-0.27	37.4

Min, minimum; Max, maximum; Std. D, standard deviation; CV, coefficient of variation.

Analysis of variance (AVOVA) combined with multiple comparisons of yield gap and influencing-factors for each variety were summarized in Table 4. Obviously, the yield gap for QY with mean value of 2.55 t/ha was significantly higher than those of GY and ZY, indicating variety had significant effects on rice yield gap. The rates of fertilizers applied to ZY was significantly higher than GY, while the mean temperature and sun hours for ZY were significantly lower than GY. Other factors among series had no obvious difference, indicating the obvious difference might be because of the difference among varieties to some extent. Additionally, yield gap among different slope positions had no significant difference (p > 0.05).

**Table 4:** Analysis of variance of influencing-factors and yield gap for each variety.

	Item	QY	GY	ZY		Item	QY	GY	ZY
**Soil**	OM(g/kg)	22.1b	27.7a	24 ab	**Climate**	Rainfall(mm)	833 a	802 a	844 a
	AvN(mg/kg)	124b	137 b	158a		Tmean(^o^C)	24.2a	24.1a	23.6b
	AvP (mg/kg)	7.5a	7.1 a	9.2a		Sun hours(h)	807 a	781a	727 b
	AvK(mg/kg)	80.8a	85.7a	86.6a		TDiff(^o^C)	8.6ab	8.4b	8.7a
**Terrain**	Elevation(m)	361 a	369 a	425 a	**Fertilizer**	N(kg/ha)	150ab	145 b	159a
	Aspect(°)	170a	154a	203 a		P_2_O_5_(kg/ha)	88.8a	80.8b	90 a
	Slope(°)	6.7a	4.9a	5.1a		K_2_O(kg/ha)	88.8a	79.8b	91.9a
	TWI	10.3a	9.3a	11.4a	**Yield**	Yield gap(t/ha)	2.55a	1.99b	1.96b

Different letters within the column represent significant difference among varieties, p<0.05.

### Correlation analysis

Yield gap for rice was negatively correlated with soil AvN while positively correlated with sun hours, P_2_O_5_, and K_2_O (Table 5). No significant correlations were observed between yield gap and other factors.

**Table 5:** Pearson correlation analysis between yield gap and influencing-factors for rice.

Item	Yield gap(t/ha)	Item	Yield gap(t/ha)
**OM (g/kg)**	-0.049	Rainfall (mm)	-0.081
**AvN (mg/kg)**	-0.309**	Tmean (^o^C)	0.115
**AvP (mg/kg)**	-0.185	Sun hours (h)	0.258*
**AvK (mg/kg)**	-0.127	TDiff (^o^C)	0.187
**Elevation (m)**	-0.081	N (kg/ha)	0.205
**Aspect (°)**	-0.039	P_2_O_5_ (kg/ha)	0.296**
**Slope (°)**	0.049	K_2_O (kg/ha)	0.284*
**TWI**	-0.109		

*and** represent significant at p<0.05 and p<0.01, respectively.

### Regression tree

**Fig 4:**
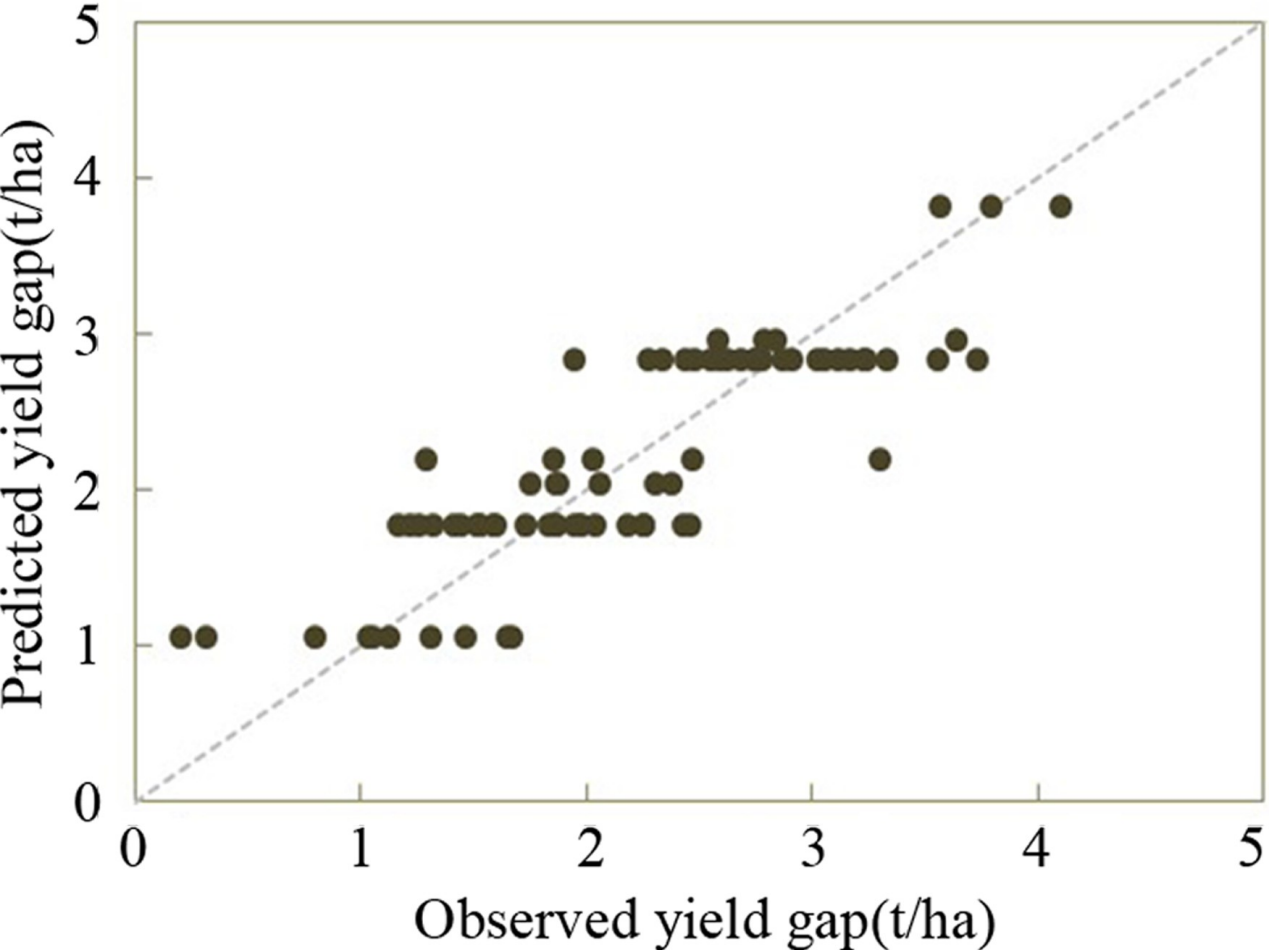
Scatter plot of observed and predicted yield gap using regression tree (The dash line is 1:1 line).

The relationship between the observations and the estimated yield gaps generated by regression tree were given in Fig 4. The model performed good with R^2^ of approximately 0.741 (p < 0.05), RMSE of 0.41 t/ha, and MAE of 0.33 t/ha. The values of R^2^, RMSE, and MAE of MLR were 0.332 (p > 0.05), 0.67 t/ha, and 0.55 t/ha. According to the statistical indicators, regression tree performed much better than MLR.

**Fig 5:**
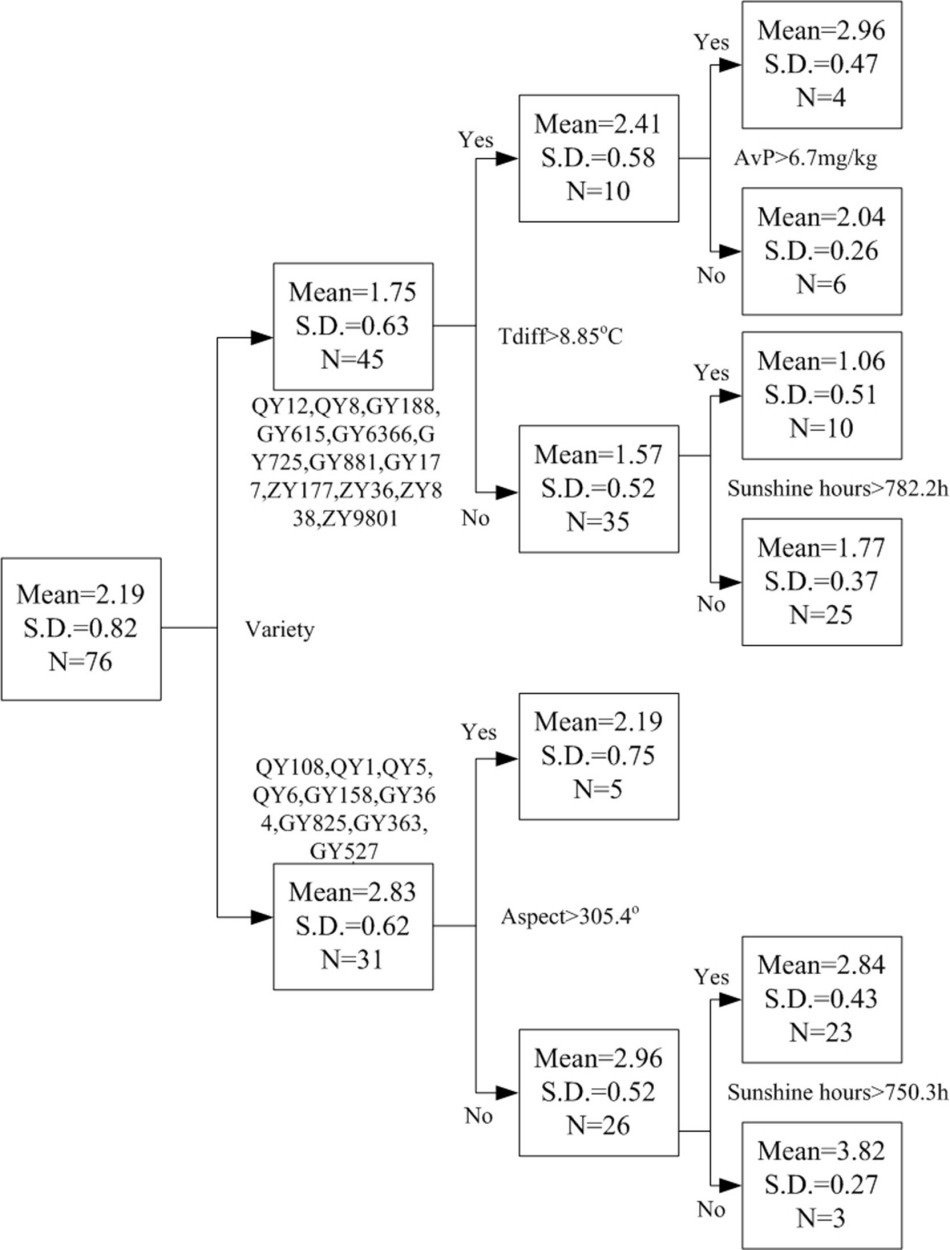
Regression tree produced by RT for rice yield gap (Mean, S.D., and N represent mean rice yield gap, standard deviation, and the total numbers of node, respectively).

The regression tree was produced by the RT method and shown in Fig 5. The root of tree was bifurcated by variety. For upper sub-tree, variety includes QY, GY, and ZY, while only partial QY and GY were separated for lower sub-tree. At the second stage, TDiff (8.85°C) and Aspect (305.4°) were applied, the yield gap was higher with larger TDiff and smaller aspect, suggesting relatively higher daily temperature difference is beneficial to improve rice yield to some extent, and larger aspect was to the disadvantage of increasing yield. At the terminal nodes of the tree, larger TDiff accompanied by higher soil AvP content could increase rice yield, while larger TDiff with higher sunshine hours had adverse influence, showing the growth of rice was affected by integrated factors. Meanwhile, GY 158 had more yield gap than QY 1 and QY 108, the possible reason is that the photonasty of rice varied with variety.

### The relative importance of factors affecting rice yield gap

**Fig 6:**
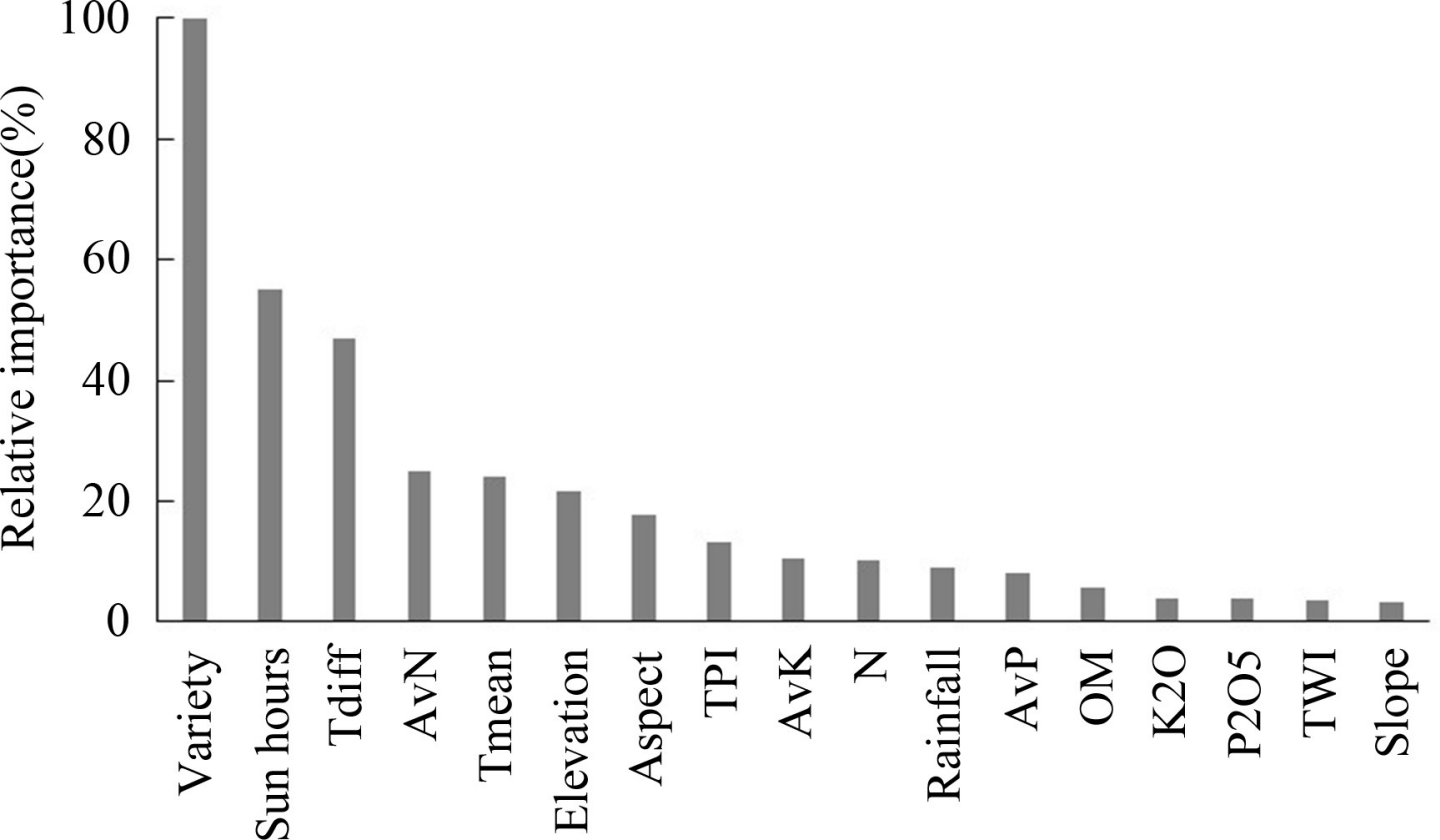
The relative importance of factors affecting rice yield gap (Tdiff: Difference between maximum and minimum temperatures, Tmean: Mean temperature, N: Nitrogenous fertilizer, K2O: Potassium fertilizer, P2O5: Phosphorus fertilizer, AvN: Available nitrogen, AvK: available potassium, AvP: Available phosphorus, OM: Organic matter).

The relative importance of factors affecting rice yield gap generated by RT was shown in Fig 6. Obviously, variety was the most important factor with relative importance of 100%. Sun hours and daily temperature difference (TDiff) were the second and the third most important factors affecting rice yield gap, respectively. Specifically, climate variables were ranked in order of sun hours > TDiff > mean temperature > rainfall. For soil properties, the rank order was AvN > AvP > AvK > OM. For terrain factors, the rank order was elevation > TPI > aspect > slope > TWI. For fertilizer factors, N fertilizer followed by K and P fertilizer played the most important role in yield gap. On average, the rank order of the studied factors was variety > climate > soil properties > terrain > fertilization.

## Discussion

In the current study, about 74.1% of rice yield gap variation was explained by using RT model. The uncertainty might be other management factors such as pest, weed, as well as plough, which were not inputted to the model. This model performed better than MLR which had lower values of R^2^ (0.332, p > 0.05) and higher values of RMSE (0.67 t/ha) and MAE (0.55 t/ha). Additionally, RT is a non-parametric method and could deal with nonlinear relationships between independent variables and response one [49]. Meanwhile, RT could provide relative importance of independent variables to dependent one. Therefore, it has been widely employed to explore the relationships between crop yield variations and soil parameters, management practices, as well as climate [30,50,51].

The presented results produced by regression tree and ANVOA suggested variety had more important influence on rice yield gap in this study area. Liu et al. [4] revealed variety updating had significant impact on improvement of rice yield and could compensate negative effect of climate change, similar results were also reported by Liu et al. [7]. Among the climate parameters, sunshine hours and daily temperature difference with higher values of relative importance were of great importance to rice yield gap. This finding was supported by previous reports [2,52,53]. For example, Xiong et al. [2] suggested sufficient sunshine was benefit to photosynthesis of leaf and transformation of carbohydrate, which increased the grain weight of rice. Liu et al. [53] reported temperature difference had direct influence on rice yield in southwest China. Higher temperature difference was beneficial to the accumulation of dry matter [8]. Another possible reason for significant effect of temperature difference to rice yield gap is ascribed to heat stress [54], which affect not only soil respiration [55], but also the absorption of rice to nutrients [56].

Although soil properties and terrain indicators had lower values of relative importance, the effects of these factors on variation in rice yield gap could not be ignored. Among soil properties, AvN played a leading role in rice yield gap variation, suggesting rice yield gap was sensitive to AvN. A possible explanation for this might be that the direct effect of AvN on grain yield by increasing the number of panicles [57] and improving stomatal conductance, net photosynthesis and transpiration [58]. Noticeably, the negative response of yield gap to AvN in Table 5 is likely due to soil inherent fertility, providing narrow space for rice yield to improve [59]. Negative correlation also was found between yield increase and soil fertility supply [60]. Among terrain variables, elevation was the most important terrain factor limiting rice yield gap. It is well-known that there is significant relationship between elevation and climate, soil property [61,62]. Therefore, elevation affects the crop yield indirectly [63].

In this paper, our result that fertilization had no significant impact on rice yield gap variation may be contrary to early reports [8,64]. For instance, Xu et al. [65] demonstrated nutrient management played the most important role in improving rice yield. On the one hand, the possible explain for our finding might be tiny difference in rates of fertilization guided by government among trials (Table 2), indicating a more precise fertilizer recommend combined with regional characteristic should be enacted to meet requirement for rice growth and decrease pollution of chemical fertilizer. On the other hand, it is likely due to spatial variation of fertilizer use efficiency caused by soil, climate, terrain among regions [66]. Therefore, in current study, although same rates of fertilizer were applied, rice yield gap varied with regions.

## Conclusion

Agricultural production system is a complicated and unique system affected by numerous factors, such as climate, soil properties, terrain. Based on detailed experimental data, results obtained from RT model suggested the selected factors could account for about 74.1% of rice yield gap variation. More detailed management factors, such as pest and weed, which were not included in present paper might become candidates to explain the remaining of variation. This paper solved a pressing problem to identify the most important factors limiting rice yield gap variation and was expected to provide reasonable advice for government in southwestern China to predict the developmental trend of rice production. Through specific analysis, we found variety and climate became the most important factors limiting improvement of rice yield, alarming the breeding researches to cultivate more new varieties with stronger adaptability to the complex environment, especially the climate warming. Meanwhile, government should enact more precise fertilization strategies to adapt to change in soil, climate, and terrain within region. Furthermore, our results also will provide valuable information to other crops in specific districts. More additional efforts should focus on systematic and detailed analysis of other factors limiting rice yield gap.

## Supporting information

S1 File Data. (ZIP)
